# Case Report: Intradural gout tophi without systemic gout symptoms

**DOI:** 10.3389/fsurg.2025.1472886

**Published:** 2025-02-10

**Authors:** Peidong Qing, Shiming Xie, Chaoqun Feng, Hongda Xu, Shengxin Zhao, Lei Zhang, Haitao Deng, Yehui Wang, Youpeng Hu

**Affiliations:** ^1^ Mianyang Orthopedic Hospital, Mianyang, China; ^2^ Hospital of Chengdu University of Traditional Chinese Medicine, Chengdu, China; ^3^ Sichuan Province Orthopedic Hospital, Chengdu, China

**Keywords:** gout tophi, spinal dura, intradural gout, hyperuricemia, lumbar scoliosis, neurological damage

## Abstract

**Background:**

Gout is a common disease; however, a gout tophus occurring within the spinal dura is exceedingly rare, with only two cases reported to date.

**Case presentation:**

We report the case of a 70-year-old female who presented with lower back pain, right radicular pain, and numbness in the perineal area. Magnetic resonance imaging and computed tomography scans revealed a calcified intradural lesion at the L3 level. The diagnosis of the lesion was not definitive because the patient had no history of gout or manifestations of systemic gout. Surgical removal of the intradural lesion followed by pathological examination confirmed gouty tophi. The postoperative recovery was good, and the patient experienced substantial relief from pain and numbness.

**Conclusion:**

This is the third documented case of gout tophi occurring within the spinal dura. In a literature review, it was found that none of these three patients with intradural gouty tophi had systemic gout manifestations or hyperuricemia, which is a crucial finding. As it is challenging to diagnose intradural gout, awareness among physicians must be increased to optimize the treatment of, and outcomes for, these patients.

## 1 Introduction

Gout is a common metabolic disorder caused by chronically elevated serum uric acid levels (1). Monosodium urate crystals primarily deposit in the peripheral joints of the extremities, where the temperature and blood circulation are too low to form gout tophi (2–4). Reports of spinal gout are rare, and an intradural gout tophus in the spinal dura is even rarer (5). The presentation of gout tophi within the spinal dura without any systemic manifestation of gout is extremely rare, with only two cases reported in the literature, and is significantly misleading and difficult for doctors to diagnose (6, 7). Herein, we report a case of gouty tophi within the spinal dura without systemic gout manifestations in a patient with degenerative lumbar scoliosis.

## 2 Case description

### 2.1 Patient information

The patient was a 70-year-old woman who had experienced lower back pain for 2 years, which was relieved with oral non-steroidal anti-inflammatory drugs. Two months prior, the pain suddenly worsened and radiated to the right buttock and lateral right lower extremity, accompanied by numbness in the perineal area. The symptoms were aggravated by coughing and sneezing. The patient had no history of spinal trauma or gout. She was 147 cm tall and weighed 38 kg, with a BMI of 17.6 kg/m^2^.

### 2.2 Diagnostic assessment

On physical examination, we noted the absence of lumbar lordosis, considerable percussion and tenderness at the L2–L5 levels, and a right supine straight leg raise angle of 55°. The pyriformis stretch and femoral nerve traction test results were negative. The patient exhibited decreased superficial sensation in the perineum and normal muscle power in the extremities. No swelling or nodes were observed in the joint examination.

**Figure 1:**
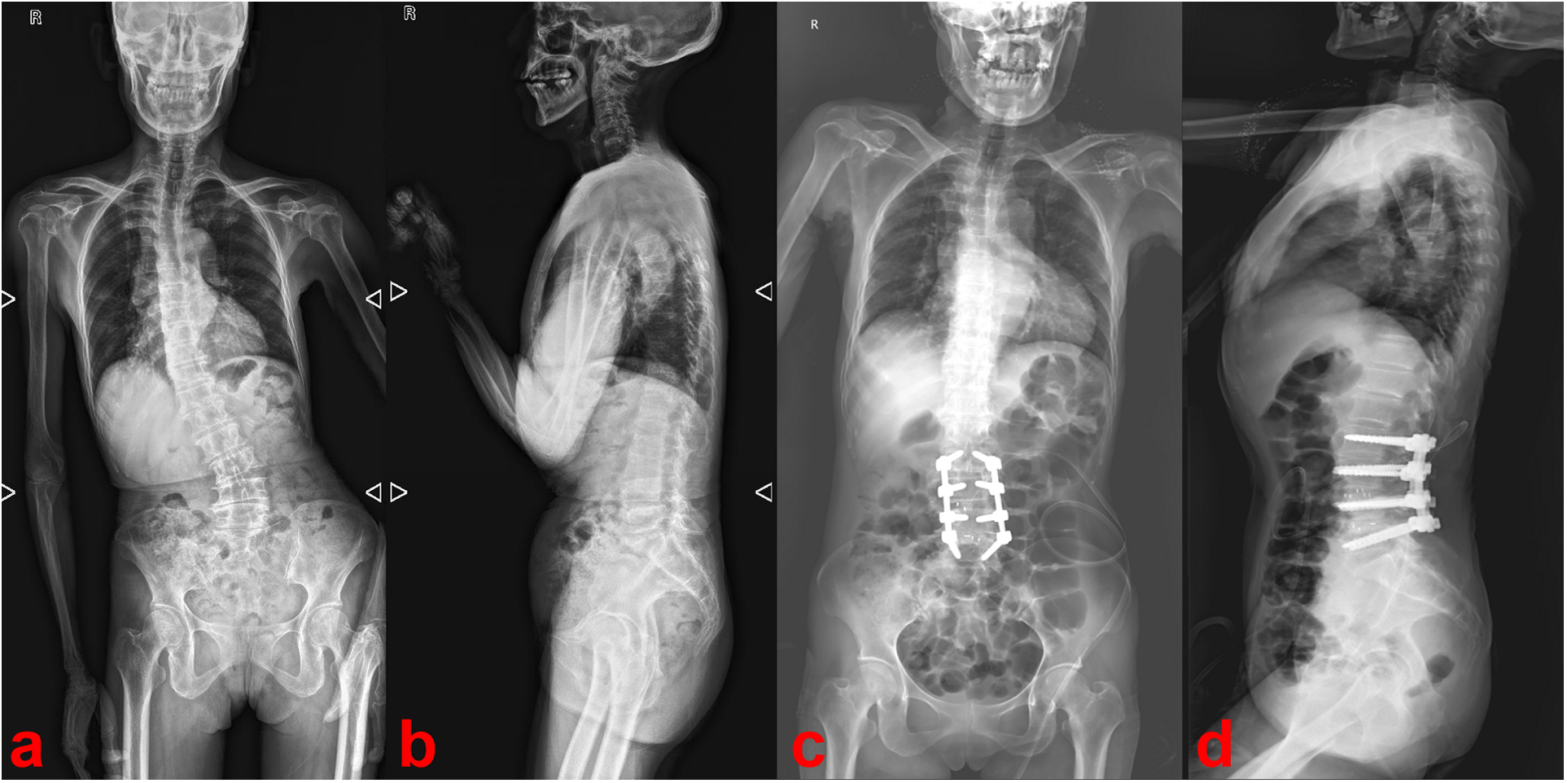
Preoperative **(a,b)** and postoperative **(c,d)** radiographs of the full-length spine.

**Figure 2:**
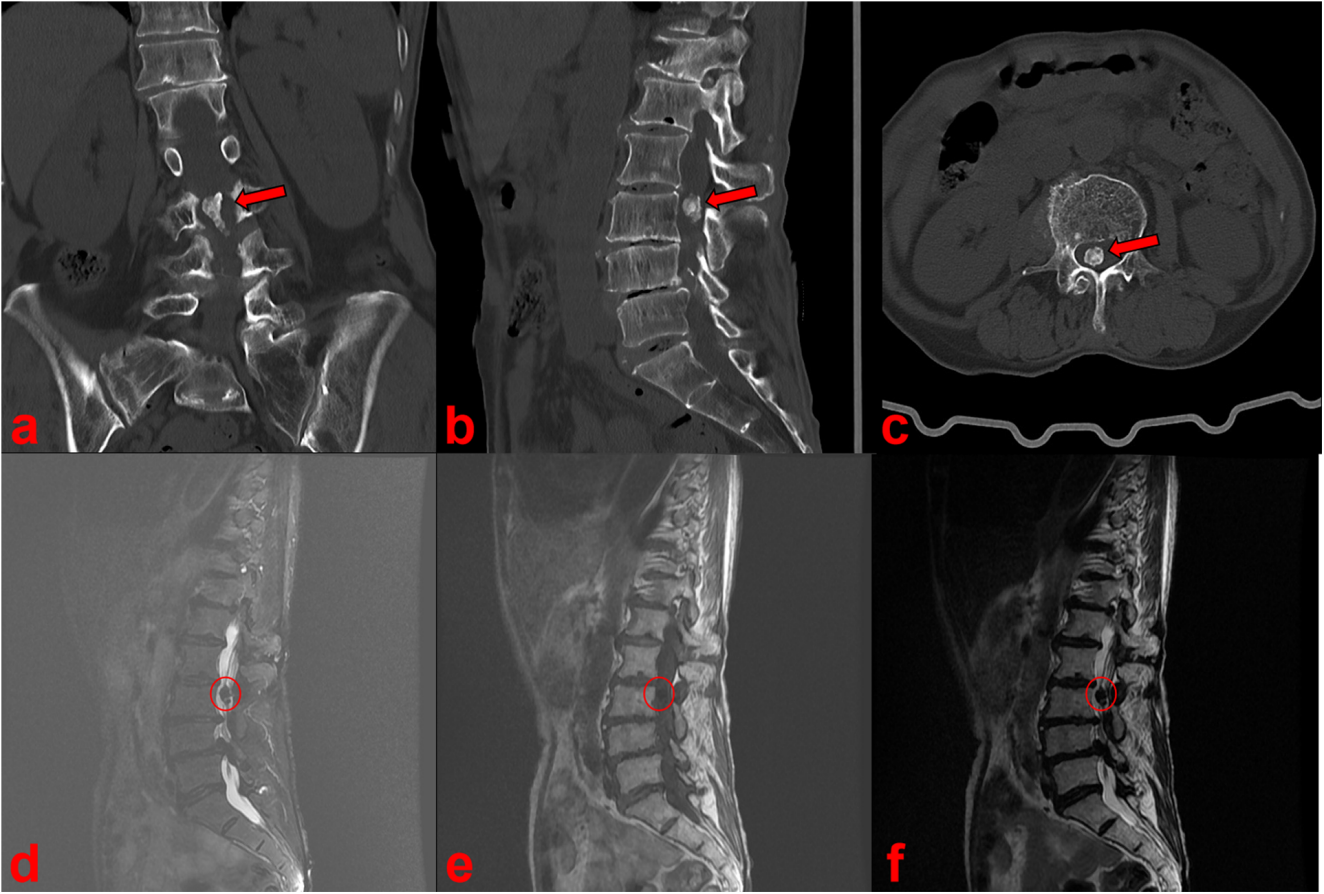
CT **(a–c)** and MRI **(d–f)** scan showed a calcified intradural lesion at the L3 vertebral level.

Radiographs of the full-length spine revealed thoracolumbar degenerative scoliosis and a pseudoslip of the L4 vertebra (Figures 1a,b). Computed tomography (CT) and magnetic resonance imaging (MRI) revealed a calcified intradural lesion at the L3 vertebral level and L3-S1 disc herniation secondary to lumbar spinal stenosis, predominantly at the L4/5 level (Figure 2). Abdominal color Doppler ultrasonography revealed a small number of urinary salt crystals in the left kidney. However, this finding was not brought to the attention of the doctors as being linked to gout.

On laboratory examination, we found that her serum uric acid level was 146 µmol/L (normal range: 155–357 µmol/L) and her serum creatinine level was 45 µmol/L (normal range: 57–97 µmol/L), both of which were low. Her glomerular filtration rate (GFR) was calculated to be 61.6 ml/min, suggesting mild renal impairment. Infection markers, such as C-reactive protein, erythrocyte sedimentation rate, and leukocyte count, were within normal ranges. The level of urea was also within the normal range.

As the patient was a woman with no history of gout and no elevated blood uric acid, and no other gout tophi were found on physical examination, the calcified intradural mass was not diagnosed as a gout tophus preoperatively but was considered more likely to be a calcified nerve sheath tumor, calcified chordoma, calcified disc prolapse, or calcified ventricular meningioma.

### 2.3 Therapeutic intervention

**Figure 3:**
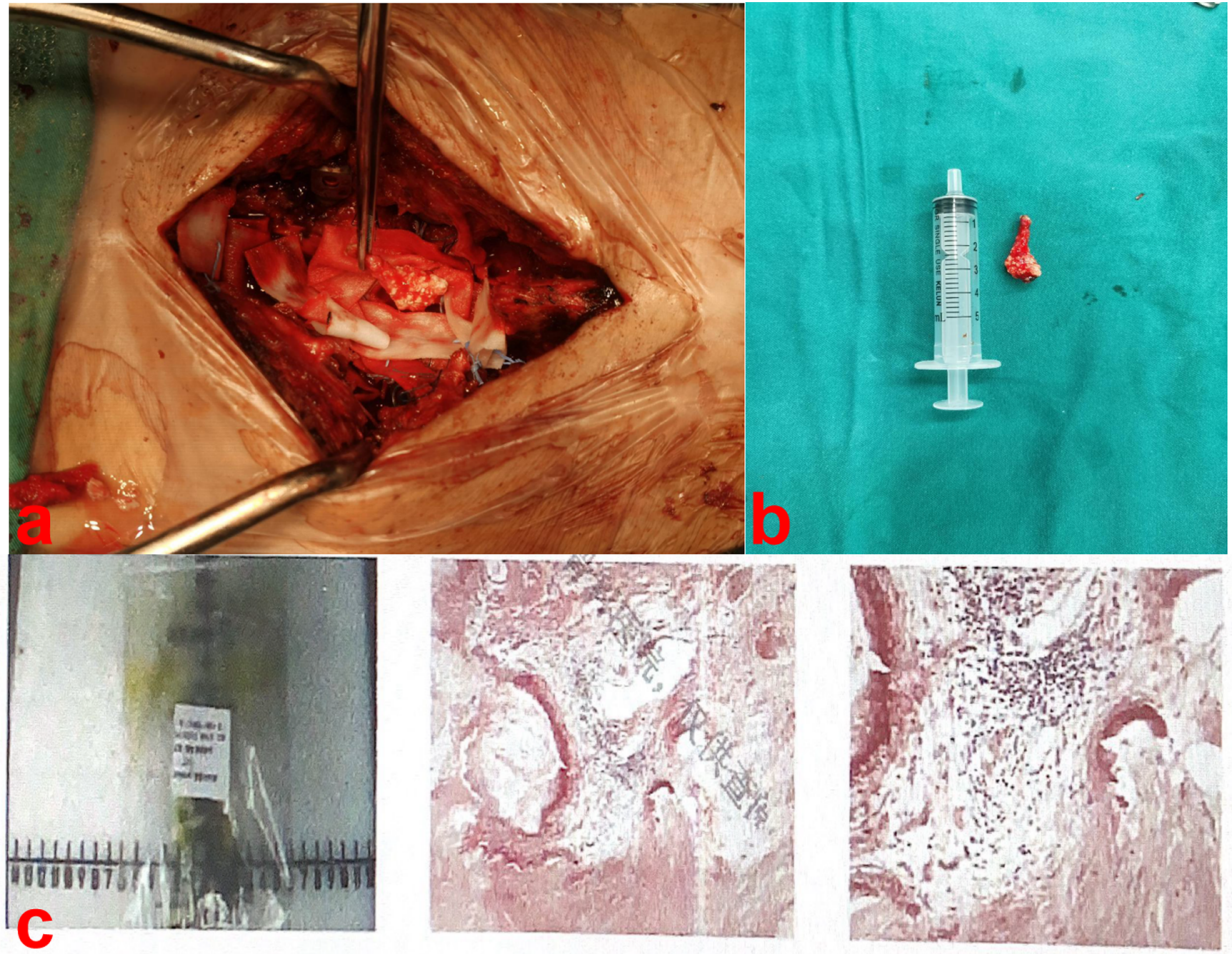
Upon opening the dura, the lesion was a white irregular crystalline mass **(a,b)**. Pathological examination showed the mass consisted of urate crystals surrounded by calcified, degenerated and necrotic nerve tissue **(c)**.

During surgery, we removed the spinous processes and lamina at the L2–L5 levels to expose the spinal canal. Upon opening the dura, the lesion was completely excised, revealing a white, irregular, crystalline mass (Figures 3a,b). Subsequently, we corrected the lumbar scoliosis deformity and released the lumbar spinal stenosis using posterior lumbar interbody fusion. The pathological examination revealed a white irregular crystalline mass consisting of urate crystals surrounded by calcified, degenerated, and necrotic nerve cells (Figure 3c).

### 2.4 Follow-up and outcomes

Postoperatively, the patient's lower back and radicular pain improved significantly and the pain intensity dropped from 7 to 3 on a 10-point visual analog scale (VAS), but numbness in the perineal area persisted. Postoperative radiographs showed the disappearance of calcification in the spinal canal and correction of the degenerative lumbar scoliosis (Figures 1c,d). The numbness in the perineal area disappeared by the sixth postoperative month.

## 3 Discussion

A global epidemiological study on gout showed that its incidence ranges from 0.3 to 6 cases per 1,000 person-years, with a prevalence ranging from 0.1% to approximately 10% (8). The primary manifestation of gout is intensely painful peripheral joint synovitis, which eventually leads to joint damage, deformity, and subcutaneous gout tophus deposition (9–11). It has been well established that hyperuricemia is the primary etiology of gout (3, 12), with risk factors including genetics, cardiovascular and inflammatory comorbidities, exposure, medications, and dietary factors (8).

Although gout can affect all the segments of the spine, it is uncommon, with only 315 cases reported as of 2023 (13, 14). The prevalence of axial gout in patients with gout is estimated to range from 14% to 35% (15–18). The prevalence of intraspinal gout has been suggested to be much higher than that previously reported as most cases are asymptomatic, resulting in insidious onset and easy misdiagnosis (19–21). In addition, the majority of gout tophi are located at the lumbar level, and men are more likely to suffer from spinal gout and have a much lower average age than women (13). Back pain is the most common symptom of spinal gout, followed by radiating pain. The treatment of choice for patients with spinal gout is usually conservative treatment with anti-inflammatory medication and urate-lowering therapy, with 88.9% of the patients improving after receiving conservative treatment (13). Surgical resection and decompression are usually initiated if a patient develops new neurological symptoms or progressive neurological damage, with 92% of patients showing improvement after surgery (13).

In 1950, Kersley reported the first case of spinal gout (22). The number of reported cases of axial gout has increased from 69 in 2009 to 315 in 2023 (13). However, only two of the 315 cases involved a gouty tophus occurring within the spinal dura (6, 7). In 2000, Paquette et al. (6) reported an intradural gout tophus in the filum terminale at the L3 vertebral level in an elderly man without systemic gout. In 2015, Willner et al. (7) similarly reported an elderly woman without systemic gout who had intradural gout tophi attached to the intradural nerve root at the L2 vertebral level. We report the third documented case of gout tophi occurring within the spinal dura at the L3 vertebral level. In the present study, the elderly woman also did not have hyperuricemia or systemic gout.

The three patients with intradural gouty tophi shared several features: (1) all complained of lower back pain, radiating pain, and progressive neurological damage; (2) all intradural gout tophi occurred in the lumbar spine and were concentrated at the L2/L3 vertebral level; (3) none had a history of gout, detectable hyperuricemia, or systemic gout manifestations; and (4) all the patients underwent surgical treatment to remove the gout tophi and recovered well after surgery.

In general, the formation of gout tophi is caused by long-term elevated and fluctuating uric acid, which causes monosodium urate crystals to deposit in the peripheral joints of the extremities where the temperature and circulation are low. However, the origin of an intradural gout tophus remains uncertain and may originate from the cerebrospinal fluid or the supply of blood vessels around the nerve root. From the analysis of the only three case reports so far, both we and Paquette found many inflammatory blood vessels and a small amount of necrotic, degenerated neural tissue surrounding the intradural gout tophi. But Willner et al. reported that their gout tophi were attached to a dorsal sensory fascicle flowing from the nerve root without any blood vessel attachment. The patient reported in this case had mild renal impairment leading to reduced uric acid excretion, which may also contribute to the development of an intradural gout tophus.

## 4 Conclusion

To the best of our knowledge, this is the third reported case of a gout tophus occurring within the spinal dura without hyperuricemia or systemic gout. Owing to the significant pain and progressive neurological damage, we performed timely surgery and the patient recovered well after surgery. As spinal gout (especially intradural gout), is difficult to diagnose, increasing awareness of intradural gout among physicians is essential to optimize treatment and outcomes for these patients. It is hoped that new imaging techniques will be available in the future to improve the diagnosis of intradural gout.

## Data Availability

The raw data supporting the conclusions of this article will be made available by the authors, without undue reservation.
